# Crystal structure and Hirshfeld surface analysis of 1-[2-(2-chloro­eth­oxy)eth­yl]-2-methyl-4-nitro-1*H*-imidazole

**DOI:** 10.1107/S2056989025005493

**Published:** 2025-06-24

**Authors:** Thaluru M. Mohan Kumar, Papegowda Bhavya, Besagarahally L. Bhaskar, Holehundi J. Shankara Prasad, Hemmige S. Yathirajan, Sean Parkin

**Affiliations:** a Department of Physical Sciences, Amrita School of Engineering, Amrita Vishwa Vidyapeetham, Bengaluru-560 035, India; b Department of Applied Sciences, New Horizon College of Engineering, Bengaluru-560 103, India; c Department of Chemistry Yuvaraja's College University of Mysore,Mysore-570 005 India; d Department of Studies in Chemistry University of Mysore, Manasagangotri Mysuru-570 006 India; e Department of Chemistry University of Kentucky,Lexington KY 40506-0055 USA; Institute of Chemistry, Chinese Academy of Sciences

**Keywords:** crystal structure, disorder, Hirshfeld surface, weak hydrogen bonds

## Abstract

Imidazoles are a widely studied class of heterocyclic compounds with significant biological and pharmacological relevance, including applications as fungicides, herbicides, and therapeutic agents. The title compound, C_8_H_12_ClN_3_O_3_ (**I**), is a structural analogue and impurity of the anti-protozoal drug metronidazole, making it valuable for mechanistic and drug development studies. Here, we report its crystal structure and Hirshfeld surface analysis. Crystals of **I** are triclinic, space-group type *P*1, with two independent mol­ecules (*A* and *B*) in the asymmetric unit, each exhibiting chloro­eth­oxy­ethyl chain disorder over two conformations. Both mol­ecules have essentially planar methyl-nitro-1*H*-imidazole cores, with conformational variation arising from the side chains. The structure lacks conventional hydrogen bonds but features several weak C—H⋯O, C—H⋯N, and C—H⋯Cl inter­actions, connecting mol­ecules into dimers and layers parallel to the *ac*-plane. Hirshfeld surface analysis reveals that the mol­ecular environments of *A* and *B* are similar and dominated by contacts involving hydrogen.

## Abstract 2

The crystal structure and a Hirshfeld surface analysis of 1-[2-(2-chloro­eth­oxy)eth­yl]-2-methyl-4-nitro-1*H*-imidazole are presented.

## 1. Chemical context

Imidazoles are a common class of heterocyclic compounds found in natural and synthetic pharmacologically active substances (Neilde *et al.*, 2014 ▸; Adamovich *et al.*, 2014 ▸; Have *et al.*, 1997 ▸), exhibiting diverse biological properties (Lombardino & Wiseman, 1974 ▸). Many imidazole derivatives act as fungicides, herbicides, plant growth regulators, therapeutic agents (Maier *et al.*, 1989 ▸), anti­cancer agents (Krezel, 1998 ▸), and bactericides (Jackson *et al.*, 2000 ▸). Recent reviews highlight the medicinal relevance of synthetic imidazole analogs (Rulhania *et al.*, 2021 ▸) and advances in imidazole-based drug development (Zhang *et al.*, 2014 ▸). Nitro-imidazoles have seen broad application in drug synthesis (Hori *et al.*, 1997 ▸), with derivatives used as radio-sensitizers, and as anti-protozoal, anti-fungal, anti-bacterial, or anti-epileptic agents (Olender *et al.*, 2009 ▸; Duan *et al.*, 2014 ▸; Sutherland *et al.*, 2010 ▸).

1-[2-(2-Chloro­eth­oxy)eth­yl]-2-methyl-4-nitro-1*H*-imidazole (**I**), C_8_H_12_ClN_3_O_3_, is an analogue of (and impurity in) the anti-protozoal drug metronidazole. Its value in drug development and mechanistic studies results from its structural similarity to metronidazole and other nitro-imidazoles. The nitro group confers distinctive chemical and biological properties, making it a promising candidate for exploring new therapies, especially against protozoal infections. In this context, we present the crystal structure and a Hirshfeld-surface analysis of **I**.
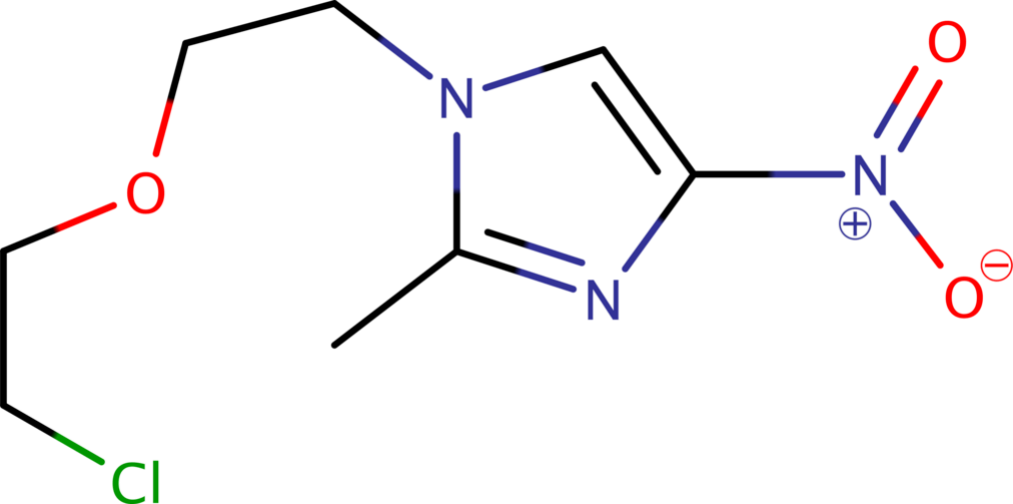


## 2. Structural commentary

**Figure 1:**
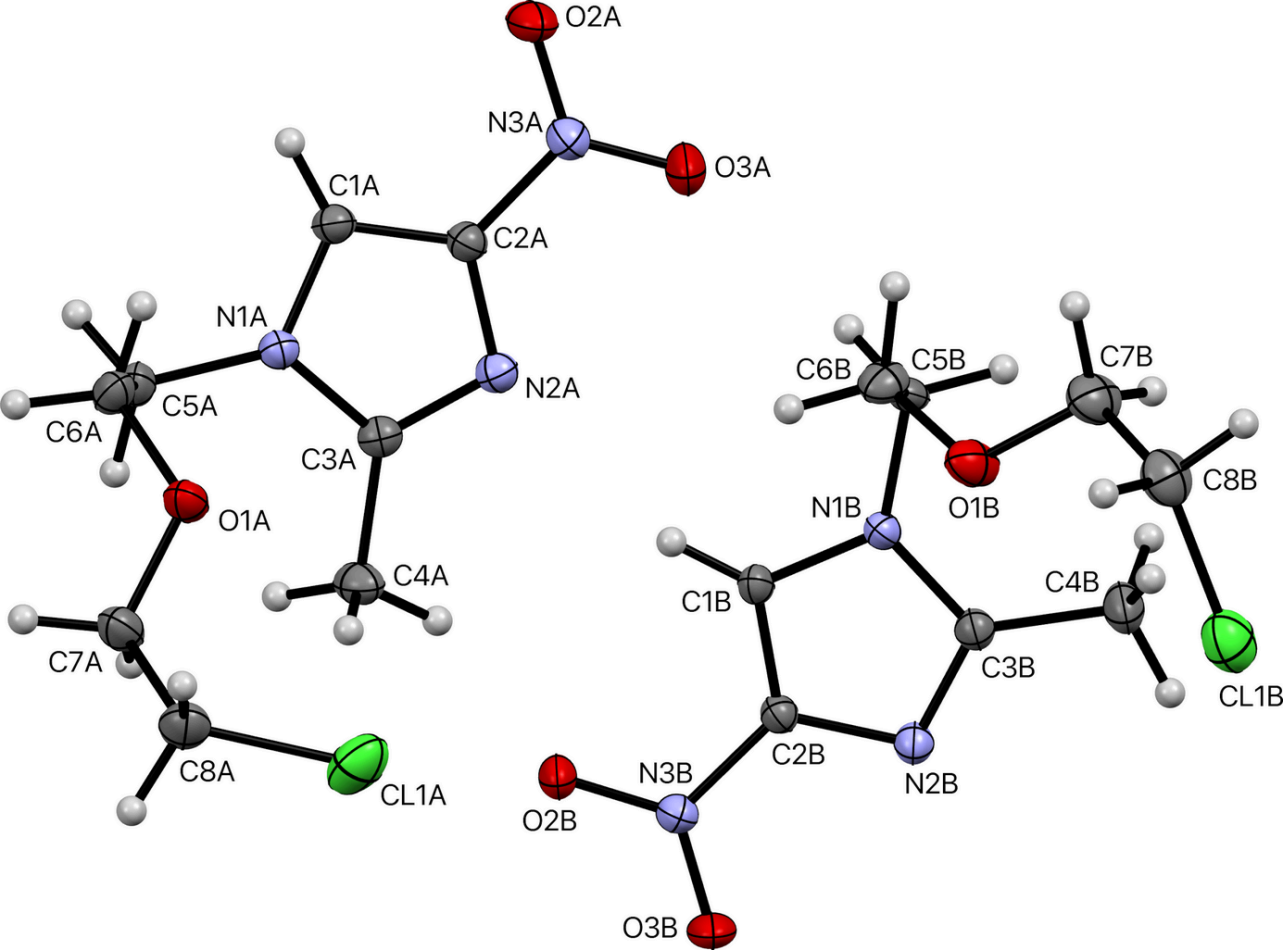
An ellipsoid plot (50% probability) of the asymmetric unit of **I**. Minor disorder components are omitted for the sake of clarity. Hydrogen atoms are shown as small white spheres of arbitrary radius.

**Figure 2:**
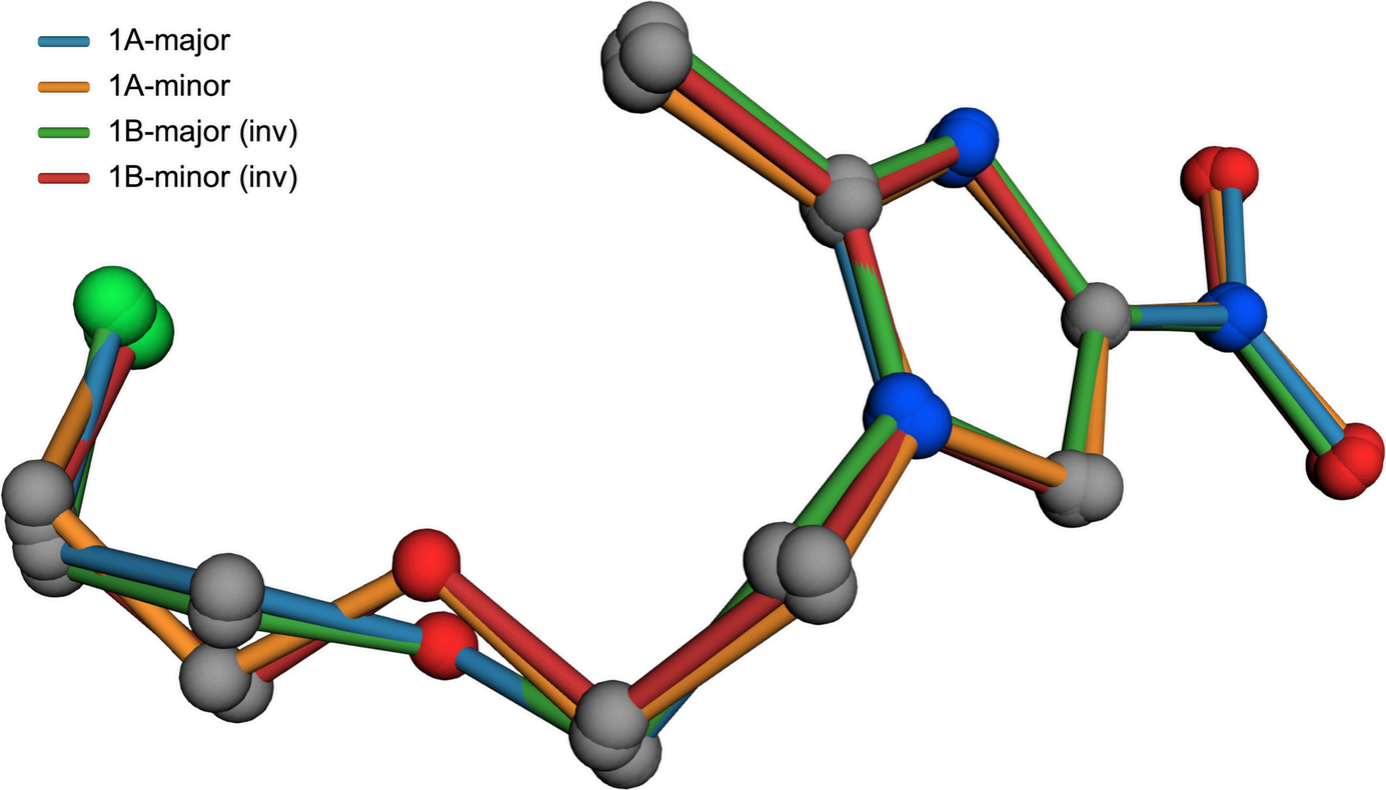
A least-squares overlay plot of the four conformations (major and minor disorder for mol­ecules *A* and *B*). Atoms are drawn with CPK colours, bond colours identify the particular conformer. Mol­ecule *B* was inverted for the optimal fit.

Crystals of **I** are triclinic, space-group type *P*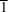, with two mol­ecules (*A* and *B*) in the asymmetric unit (*Z*′ = 2). Chemically, the mol­ecules comprise an imidazole ring substituted with *N*-nitro, methyl, and chloro­eth­oxy­ethyl groups (see Scheme and Fig. 1 ▸). The chloro­eth­oxy­ethyl chains of both independent mol­ecules are each disordered over two conformations with refined major:minor occupancies of 0.7256 (4):0.2744 (4) and 0.6384 (4):0.3616 (4) for mol­ecules *A* and *B* respectively. Thus, there are four separate conformations. However, as is clear from a least-squares overlay plot (Fig. 2 ▸), the two major conformers are very similar, as are the two minor conformers. This similarity prompted us to test whether the structure was simpler at higher temperatures, either by resolution of the disorder, or by transition to a *Z*′ = 1 structure. No such changes were apparent up to 250 K.

The methyl-nitro-1*H*-imidazole moieties are largely planar [r.m.s. deviation = 0.0242 Å (*A*), 0.0584 Å (*B*)], with maximum deviation at atoms O3A [0.0456 (17) Å] and O3B [0.1144 (17) Å], resulting from slight twists of the nitro groups [dihedrals with the imidazole ring are 2.75 (13)° and 5.64 (6)° in *A* and *B*, respectively. The overall geometry of the mol­ecules results from the relative orientations of the planar moieties with the chloro­eth­oxy­ethyl chains, whose conformations result from torsions about the N1—C5, C5—C6, C6—O1, O1—C7, and C7—C8 bonds, as qu­anti­fied in Table 1 ▸ and shown in the overlay (Fig. 2 ▸).

## 3. Supra­molecular features

**Figure 3:**
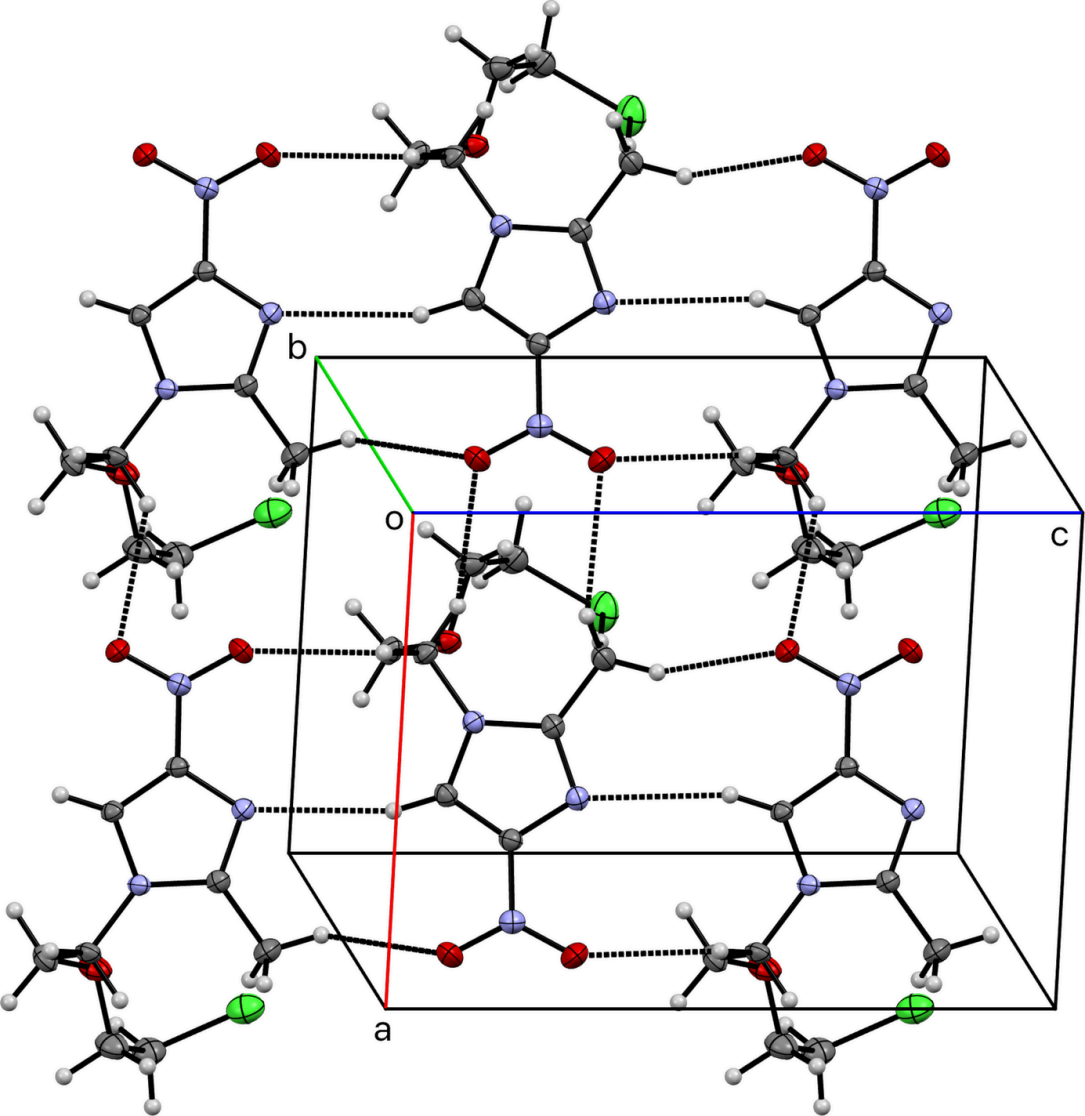
A partial packing plot of **I** viewed normal to the *ac* plane. A selection of the weak hydrogen bonds listed in Table 2 ▸ are drawn as dotted lines, highlighting ring motifs with graph-set notation 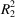
(9).

There are no conventional hydrogen bonds in **I**, but a number of weak hydrogen-bond-type contacts are flagged by *SHELXL* as ‘potential hydrogen bonds’. These are listed in Table 2 ▸ for major and minor disorder components of both *A* and *B*. One such weak inter­action is strictly *intra*-mol­ecular, namely C4*A*—H4*AA*⋯Cl1*A* [*d_D–A_* = 3.888 (2) Å], enclosing an *S*(10) motif (Etter *et al.*, 1990 ▸). For the sake of simplicity, considering just the major disorder components within the asymmetric unit, three *inter*-mol­ecular contacts C4*A*—H4*AB*⋯O2*B*, C1*B*—H1*B*⋯N2*A*, and C5*B*—H5*B*1⋯O3*A* connect the independent mol­ecules into dimers, enclosing two different 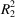
(9) ring motifs, which combine with symmetry equivalents to link the mol­ecules into layers parallel to the *ac* plane, as shown in Fig. 3 ▸. Additional contacts between layers build up the full three-dimensional structure.

**Figure 4:**
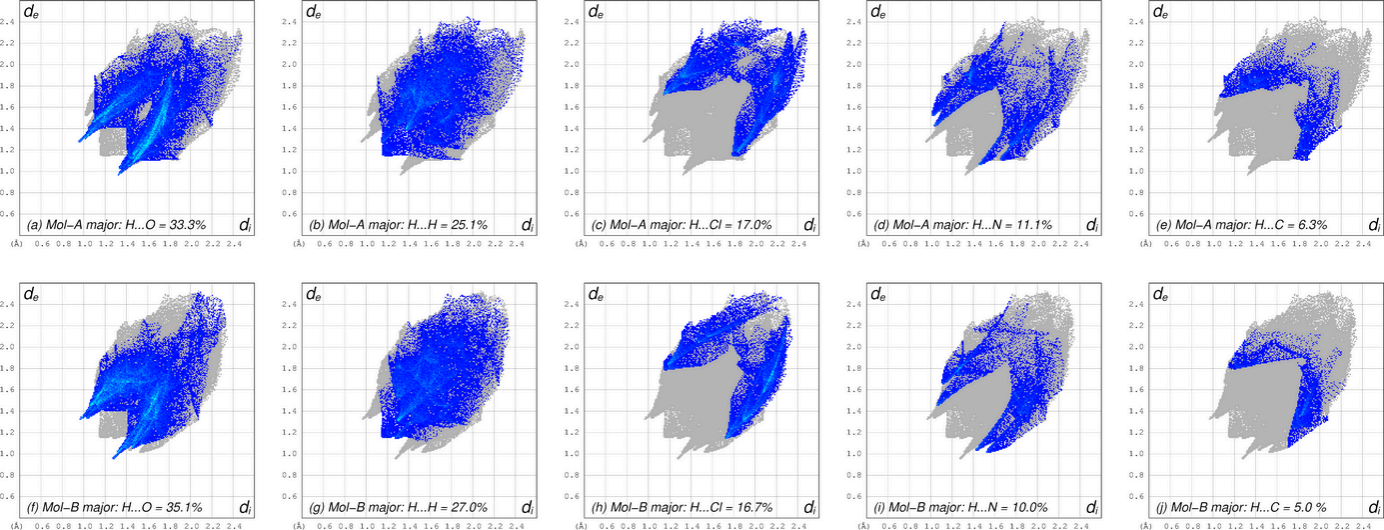
Hirshfeld surface two-dimensional-fingerprint plots calculated individually for the major disorder components in **I**. The panels are arranged in vertical pairs for (*a*,*f*) H⋯O contacts (33.3%, 35.1% for *A* and *B*, respectively, (*b*,*g*) H⋯H (25.1%, 27.0%), (*c*,*h*) H⋯Cl (17%, 16.7%), (*d*,*i*) H⋯N (11.1%, 10.0%), (*e*,*j*) H⋯C (6.2%, 5.5%), showing the similar environments for both independent mol­ecules.

A Hirshfeld surface analysis conducted using *CrystalExplorer21* (Spackman *et al.*, 2021 ▸) calculated independently for mol­ecules *A* and *B* (major components only) indicate that the environment of each mol­ecule is similar and that the vast majority of inter­molecular contacts involve hydrogen (92.8% for *A*, 93.8% for *B*). These results are summarized in the 2D-fingerprint plots shown in Fig. 4 ▸.

## 4. Database survey

A search of the Cambridge Structural Database (CSD, v5.46, Nov. 2024; Groom *et al.*, 2016 ▸) using a search fragment consisting of 2-methyl-4-nitro-1*H*-imidazole and *X* = ‘any group’ attached to the equivalent of N1(*A*/*B*) resulted in 116 hits. Searches with ‘-C-*X*’ and ‘-C-C-*X*’ at that position gave 52 and 33 matches, respectively, while a search with ‘-C-C-O-*X*’ returned six hits, two of which were duplicates. Of the four unique structures, CADDUJ (Yu *et al.*, 2015 ▸) has a tetra­phenyl Zn(EtOH)-porphyrinato group attached at the equivalent of O1(*A*/*B*). Entry IFOSUN (Zama *et al.*, 2013 ▸) has a methyl ester attached at O1(*A*/*B*) and KUZVUX (Wang *et al.*, 2010 ▸) has an ethyl ester group. The remaining refcode, NOBVIJ (Skupin *et al.*, 1997 ▸), has a methyl ester at O1(*A*/*B*) and a chloro­methyl group attached to C6(*A*/*B*).

## 5. Synthesis and crystallization

The sample of **I** was synthesized as per the literature procedure of Kaifez *et al.* (1968 ▸). In brief, direct alkyl­ation of 2-methyl-4-nitro­imidazole led to the product, which was then purified by column chromatography (silica gel, ethyl acetate/hexane system) and recrystallized from ethyl­acetate by slow evaporation (m.p.: 383–385 K).

## 6. Refinement

Crystal data, data collection and structure refinement details are summarized in Table 3 ▸. Hydrogen atoms were found in difference-Fourier maps, but subsequently included in the refinement using riding models, with constrained distances set to 0.95 Å (C*sp*^2^—H), 0.98 Å (*R*CH_3_) and 0.99 Å (*R*_2_CH_2_). *U*_iso_(H) parameters were set to values of either 1.2*U*_eq_ or 1.5*U*_eq_ (*R*CH_3_ only) of the attached atom. To ensure satisfactory refinement for the disordered chains in the structure, a combination of constraints and restraints were used. The constraints (*SHELXL* command EADP) were used to equalize displacement parameters of overlapping disordered atoms. Restraints were used to maintain the fidelity of the disordered chains (*SHELXL* commands SAME, SADI, SIMU, and RIGU).

## Supplementary Material

Crystal structure: contains datablock(s) I, global. DOI: 10.1107/S2056989025005493/nx2027sup1.cif

Structure factors: contains datablock(s) I. DOI: 10.1107/S2056989025005493/nx2027Isup2.hkl

CCDC reference: 2465512

Additional supporting information:  crystallographic information; 3D view; checkCIF report

## Figures and Tables

**Table 1:** Conformation-defining torsion angles (°) in **I**

Torsion angle	*A* (major)	*A* (minor)	*B* (major)	*B* (minor)
C1—N1—C5—C6	−76.1 (7)	−92.7 (19)	71.4 (9)	88.2 (13)
N1—C5—C6—O1	−65.6 (8)	−64 (2)	63.8 (10)	67.1 (16)
C5—C6—O1—C7	−81.8 (5)	−169.2 (10)	88.4 (5)	176.3 (8)
C6—O1—C7—C8	−165.1 (3)	168.8 (7)	166.9 (3)	−162.7 (6)
O1—C7—C8—Cl1	−66.8 (2)	68.1 (6)	68.2 (4)	−69.9 (7)

**Table 2:** Hydrogen-bond geometry (Å, °)

*D*—H⋯*A*	*D*—H	H⋯*A*	*D*⋯*A*	*D*—H⋯*A*
C1*A*—H1*A*⋯N2*B*^i^	0.95	2.61	3.5144 (15)	160
C4*A*—H4*AA*⋯Cl1*A*	0.98	2.91	3.888 (2)	172
C4*A*—H4*AA*⋯O1*C*	0.98	2.39	3.024 (4)	122
C4*A*—H4*AB*⋯O2*B*	0.98	2.34	3.2626 (14)	157
C5*A*—H5*A*1⋯O2*A*^ii^	0.99	2.65	3.562 (10)	153
C5*A*—H5*A*2⋯O3*B*^i^	0.99	2.51	3.202 (10)	127
C7*A*—H7*A*2⋯Cl1*B*^iii^	0.99	2.96	3.778 (3)	141
C8*A*—H8*A*1⋯O1*B*^iv^	0.99	2.42	3.338 (3)	153
C8*A*—H8*A*1⋯Cl1*B*^iv^	0.99	2.99	3.725 (3)	132
C8*A*—H8*A*2⋯N2*B*^iv^	0.99	2.63	3.419 (3)	137
C5*C*—H5*C*2⋯O3*B*^i^	0.99	2.31	3.16 (3)	144
C1*B*—H1*B*⋯N2*A*	0.95	2.65	3.5495 (14)	157
C4*B*—H4*BB*⋯O2*A*^v^	0.98	2.45	3.2622 (15)	140
C4*B*—H4*BC*⋯O1*D*	0.98	2.38	3.124 (3)	133
C5*B*—H5*B*1⋯O3*A*	0.99	2.53	3.199 (12)	124
C5*B*—H5*B*1⋯O2*B*^vi^	0.99	2.60	3.439 (10)	142
C5*B*—H5*B*2⋯O2*B*^vii^	0.99	2.60	3.379 (10)	135
C8*B*—H8*B*2⋯O1*A*^viii^	0.99	2.39	3.292 (5)	151
C5*D*—H5*D*1⋯O3*A*	0.99	2.53	3.26 (2)	130
C5*D*—H5*D*1⋯O2*B*^vi^	0.99	2.52	3.345 (19)	141
C5*D*—H5*D*2⋯O2*B*^vii^	0.99	2.61	3.297 (17)	127
C7*D*—H7*D*2⋯Cl1*C*^vii^	0.99	2.81	3.742 (6)	157

Symmetry codes: (i) 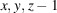; (ii) 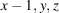; (iii) 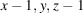; (iv) 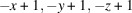; (v) 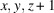; (vi) 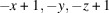; (vii) 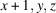; (viii) 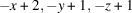.

**Table 3:** Experimental details

Crystal data	
Chemical formula	C_8_H_12_ClN_3_O_3_
*M*_r_	233.66
Crystal system, space group	Triclinic, *P* 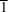
Temperature (K)	100
*a*, *b*, *c* (Å)	8.5810 (4), 11.1448 (7), 11.5757 (5)
α, β, γ (°)	98.662 (2), 93.164 (2), 103.444 (2)
*V* (Å^3^)	1059.71 (10)
*Z*	4
Radiation type	Mo *K*α
μ (mm^−1^)	0.35
Crystal size (mm)	0.25 × 0.21 × 0.13

Data collection	
Diffractometer	Bruker D8 Venture dual source
Absorption correction	Multi-scan (*SADABS*; Krause *et al.*, 2015 ▸)
*T*_min_, *T*_max_	0.892, 0.959
No. of measured, independent and observed [*I* > 2σ(*I*)] reflections	34130, 4879, 4337
*R*_int_	0.025
(sin θ/λ)_max_ (Å^−1^)	0.651

Refinement	
*R*[*F*^2^ > 2σ(*F*^2^)], *wR*(*F*^2^), *S*	0.031, 0.076, 1.06
No. of reflections	4879
No. of parameters	365
No. of restraints	416
H-atom treatment	H-atom parameters constrained
Δρ_max_, Δρ_min_ (e Å^−3^)	0.51, −0.35

Computer programs: *APEX5* (Bruker, 2023 ▸), *SHELXT* (Sheldrick, 2015*a* ▸), *SHELXL2019/3* (Sheldrick, 2015*b* ▸), *Mercury* (Macrae *et al.*, 2020 ▸), *over-lay.py* (Parkin, 2025 ▸), *SHELX* (Sheldrick, 2008 ▸) and *publCIF* (Westrip 2010 ▸).

## supplementary crystallographic information

### 1-[2-(2-Chloroethoxy)ethyl]-2-methyl-4-nitro-1H-imidazole Crystal data

**Table:**

C_8_H_12_ClN_3_O_3_	*Z* = 4
*M**_r_* = 233.66	*F*(000) = 488
Triclinic, *P*1	*D*_x_ = 1.465 Mg m^−^^3^
*a* = 8.5810 (4) Å	Mo *K*α radiation, λ = 0.71073 Å
*b* = 11.1448 (7) Å	Cell parameters from 9978 reflections
*c* = 11.5757 (5) Å	θ = 2.7–27.6°
α = 98.662 (2)°	µ = 0.35 mm^−^^1^
β = 93.164 (2)°	*T* = 100 K
γ = 103.444 (2)°	Solvent-rounded block, colourless
*V* = 1059.71 (10) Å^3^	0.25 × 0.21 × 0.13 mm

### 1-[2-(2-Chloroethoxy)ethyl]-2-methyl-4-nitro-1H-imidazole Data collection

**Table:**

Bruker D8 Venture dual source diffractometer	4879 independent reflections
Radiation source: microsource	4337 reflections with *I* > 2σ(*I*)
Detector resolution: 7.41 pixels mm^-1^	*R*_int_ = 0.025
φ and ω scans	θ_max_ = 27.6°, θ_min_ = 1.9°
Absorption correction: multi-scan (*SADABS*; Krause *et al*., 2015)	*h* = −10→11
*T*_min_ = 0.892, *T*_max_ = 0.959	*k* = −14→14
34130 measured reflections	*l* = −15→14

### 1-[2-(2-Chloroethoxy)ethyl]-2-methyl-4-nitro-1H-imidazole Refinement

**Table:**

Refinement on *F*^2^	Primary atom site location: structure-invariant direct methods
Least-squares matrix: full	Secondary atom site location: difference Fourier map
*R*[*F*^2^ > 2σ(*F*^2^)] = 0.031	Hydrogen site location: difference Fourier map
*wR*(*F*^2^) = 0.076	H-atom parameters constrained
*S* = 1.06	*w* = 1/[σ^2^(*F*_o_^2^) + (0.0258*P*)^2^ + 0.4798*P*] where *P* = (*F*_o_^2^ + 2*F*_c_^2^)/3
4879 reflections	(Δ/σ)_max_ < 0.001
365 parameters	Δρ_max_ = 0.51 e Å^−^^3^
416 restraints	Δρ_min_ = −0.35 e Å^−^^3^

### 1-[2-(2-Chloroethoxy)ethyl]-2-methyl-4-nitro-1H-imidazole Special details

**Table:**

Experimental. The crystal was mounted using polyisobutene oil on the tip of a fine glass fibre, which was fastened in a copper mounting pin with electrical solder. It was placed directly into the cold gas stream of a liquid-nitrogen based cryostat (Hope, 1994; Parkin & Hope, 1998). Diffraction data were collected with the crystal at 100K.
Geometry. All esds (except the esd in the dihedral angle between two l.s. planes) are estimated using the full covariance matrix. The cell esds are taken into account individually in the estimation of esds in distances, angles and torsion angles; correlations between esds in cell parameters are only used when they are defined by crystal symmetry. An approximate (isotropic) treatment of cell esds is used for estimating esds involving l.s. planes.
Refinement. Refinement progress was checked using *Platon* (Spek, 2020) and by an *R*-tensor (Parkin, 2000). The final model was further checked with the IUCr utility *checkCIF*.

### 1-[2-(2-Chloroethoxy)ethyl]-2-methyl-4-nitro-1H-imidazole Fractional atomic coordinates and isotropic or equivalent isotropic displacement parameters (Å^2^)

**Table:**

	*x*	*y*	*z*	*U*_iso_*/*U*_eq_	Occ. (<1)
O2A	0.92119 (11)	0.09620 (9)	0.10635 (8)	0.0291 (2)	
O3A	0.92893 (11)	0.11525 (10)	0.29564 (8)	0.0349 (2)	
N1A	0.47096 (11)	0.15624 (9)	0.13212 (8)	0.01687 (19)	
N2A	0.62164 (11)	0.14669 (9)	0.29175 (8)	0.01833 (19)	
N3A	0.86024 (12)	0.11372 (10)	0.19955 (9)	0.0218 (2)	
C1A	0.61200 (13)	0.13743 (10)	0.09384 (10)	0.0187 (2)	
H1A	0.641682	0.129776	0.015705	0.022*	
C2A	0.70087 (13)	0.13209 (10)	0.19353 (10)	0.0178 (2)	
C3A	0.48135 (13)	0.16128 (10)	0.25182 (9)	0.0173 (2)	
C4A	0.35045 (15)	0.18143 (12)	0.32518 (11)	0.0245 (2)	
H4AA	0.344755	0.269030	0.332592	0.037*	
H4AB	0.372346	0.161678	0.403229	0.037*	
H4AC	0.247736	0.126885	0.288206	0.037*	
C5A	0.3362 (11)	0.1720 (6)	0.0572 (9)	0.0202 (4)	0.726 (4)
H5A1	0.237703	0.158758	0.099079	0.024*	0.726 (4)
H5A2	0.316384	0.108581	−0.015167	0.024*	0.726 (4)
C6A	0.3727 (5)	0.3019 (4)	0.0251 (3)	0.0229 (6)	0.726 (4)
H6A1	0.475029	0.316384	−0.012149	0.027*	0.726 (4)
H6A2	0.286365	0.306822	−0.033069	0.027*	0.726 (4)
O1A	0.38532 (18)	0.39806 (13)	0.12420 (15)	0.0220 (4)	0.726 (4)
C7A	0.2349 (2)	0.41695 (19)	0.15685 (16)	0.0259 (5)	0.726 (4)
H7A1	0.180335	0.347938	0.196203	0.031*	0.726 (4)
H7A2	0.165106	0.417948	0.086170	0.031*	0.726 (4)
C8A	0.2632 (3)	0.5393 (2)	0.2385 (2)	0.0274 (5)	0.726 (4)
H8A1	0.158339	0.558782	0.252617	0.033*	0.726 (4)
H8A2	0.326692	0.606367	0.200905	0.033*	0.726 (4)
Cl1A	0.36807 (19)	0.53736 (14)	0.37668 (12)	0.0386 (2)	0.726 (4)
C5C	0.341 (3)	0.1759 (15)	0.054 (2)	0.0202 (4)	0.274 (4)
H5C1	0.236152	0.137875	0.081366	0.024*	0.274 (4)
H5C2	0.345245	0.132358	−0.026044	0.024*	0.274 (4)
C6C	0.3512 (15)	0.3136 (10)	0.0509 (8)	0.0217 (15)	0.274 (4)
H6C1	0.457012	0.354212	0.027037	0.026*	0.274 (4)
H6C2	0.265792	0.322112	−0.006061	0.026*	0.274 (4)
O1C	0.3310 (6)	0.3704 (3)	0.1662 (4)	0.0233 (9)	0.274 (4)
C7C	0.3083 (7)	0.4938 (5)	0.1727 (4)	0.0267 (11)	0.274 (4)
H7C1	0.225414	0.495346	0.110339	0.032*	0.274 (4)
H7C2	0.410135	0.552217	0.161089	0.032*	0.274 (4)
C8C	0.2564 (7)	0.5327 (6)	0.2901 (5)	0.0242 (12)	0.274 (4)
H8C1	0.161515	0.468239	0.303757	0.029*	0.274 (4)
H8C2	0.222764	0.611874	0.290270	0.029*	0.274 (4)
Cl1C	0.4080 (5)	0.5545 (4)	0.4069 (3)	0.0406 (6)	0.274 (4)
O2B	0.31302 (10)	0.11153 (8)	0.58764 (7)	0.02304 (18)	
O3B	0.32527 (10)	0.15036 (9)	0.77777 (7)	0.0271 (2)	
N1B	0.79607 (11)	0.14491 (9)	0.64445 (8)	0.01650 (19)	
N2B	0.64632 (11)	0.15332 (9)	0.79508 (8)	0.01790 (19)	
N3B	0.38786 (11)	0.13284 (9)	0.68559 (8)	0.01845 (19)	
C1B	0.64369 (13)	0.13249 (10)	0.59632 (9)	0.0163 (2)	
H1B	0.607010	0.122179	0.515661	0.020*	
C2B	0.55580 (13)	0.13829 (10)	0.69080 (9)	0.0160 (2)	
C3B	0.79288 (13)	0.15741 (10)	0.76422 (10)	0.0177 (2)	
C4B	0.93855 (15)	0.17517 (13)	0.84634 (11)	0.0267 (3)	
H4BA	0.982002	0.100900	0.832161	0.040*	
H4BB	0.910085	0.187629	0.927358	0.040*	
H4BC	1.019765	0.248707	0.833718	0.040*	
C5B	0.9357 (11)	0.1542 (6)	0.5756 (11)	0.0193 (8)	0.638 (4)
H5B1	0.910198	0.085226	0.507494	0.023*	0.638 (4)
H5B2	1.027707	0.143070	0.624673	0.023*	0.638 (4)
C6B	0.9854 (6)	0.2777 (4)	0.5312 (4)	0.0233 (8)	0.638 (4)
H6B1	1.073322	0.274029	0.479890	0.028*	0.638 (4)
H6B2	0.892957	0.289801	0.483202	0.028*	0.638 (4)
O1B	1.03761 (19)	0.38096 (16)	0.62241 (18)	0.0281 (5)	0.638 (4)
C7B	1.2020 (3)	0.4077 (3)	0.6584 (2)	0.0312 (6)	0.638 (4)
H7B1	1.263984	0.406201	0.588835	0.037*	0.638 (4)
H7B2	1.223886	0.343287	0.702940	0.037*	0.638 (4)
C8B	1.2537 (6)	0.5338 (5)	0.7340 (4)	0.0293 (9)	0.638 (4)
H8B1	1.220284	0.596075	0.691951	0.035*	0.638 (4)
H8B2	1.372571	0.557584	0.747663	0.035*	0.638 (4)
Cl1B	1.1692 (2)	0.5392 (2)	0.87550 (19)	0.0367 (3)	0.638 (4)
C5D	0.937 (2)	0.1368 (12)	0.580 (2)	0.0193 (8)	0.362 (4)
H5D1	0.904352	0.077503	0.504957	0.023*	0.362 (4)
H5D2	1.015649	0.106792	0.627016	0.023*	0.362 (4)
C6D	1.0118 (11)	0.2649 (8)	0.5563 (6)	0.0236 (13)	0.362 (4)
H6D1	1.098517	0.261355	0.503766	0.028*	0.362 (4)
H6D2	0.929752	0.298498	0.517142	0.028*	0.362 (4)
O1D	1.0767 (3)	0.3445 (2)	0.6658 (2)	0.0178 (6)	0.362 (4)
C7D	1.1612 (5)	0.4697 (4)	0.6524 (3)	0.0271 (9)	0.362 (4)
H7D1	1.083704	0.520077	0.636796	0.033*	0.362 (4)
H7D2	1.229548	0.466398	0.586434	0.033*	0.362 (4)
C8D	1.2633 (12)	0.5263 (9)	0.7665 (6)	0.0341 (17)	0.362 (4)
H8D1	1.338712	0.605825	0.758187	0.041*	0.362 (4)
H8D2	1.327000	0.468248	0.788573	0.041*	0.362 (4)
Cl1D	1.1350 (4)	0.5536 (4)	0.8748 (4)	0.0367 (3)	0.362 (4)

### 1-[2-(2-Chloroethoxy)ethyl]-2-methyl-4-nitro-1H-imidazole Atomic displacement parameters (Å^2^)

**Table:**

	*U*^11^	*U*^22^	*U*^33^	*U*^12^	*U*^13^	*U*^23^
O2A	0.0239 (4)	0.0449 (6)	0.0233 (4)	0.0149 (4)	0.0088 (3)	0.0084 (4)
O3A	0.0228 (5)	0.0607 (7)	0.0221 (5)	0.0145 (4)	−0.0032 (4)	0.0051 (4)
N1A	0.0171 (4)	0.0174 (4)	0.0162 (4)	0.0051 (4)	0.0012 (3)	0.0017 (3)
N2A	0.0196 (5)	0.0188 (5)	0.0170 (4)	0.0052 (4)	0.0027 (4)	0.0030 (4)
N3A	0.0174 (5)	0.0271 (5)	0.0209 (5)	0.0055 (4)	0.0017 (4)	0.0041 (4)
C1A	0.0190 (5)	0.0199 (5)	0.0175 (5)	0.0056 (4)	0.0037 (4)	0.0015 (4)
C2A	0.0156 (5)	0.0191 (5)	0.0186 (5)	0.0044 (4)	0.0023 (4)	0.0019 (4)
C3A	0.0188 (5)	0.0154 (5)	0.0177 (5)	0.0040 (4)	0.0030 (4)	0.0031 (4)
C4A	0.0249 (6)	0.0312 (6)	0.0222 (6)	0.0123 (5)	0.0076 (5)	0.0086 (5)
C5A	0.0183 (8)	0.0229 (7)	0.0180 (7)	0.0063 (6)	−0.0032 (6)	−0.0011 (5)
C6A	0.0271 (15)	0.0249 (11)	0.0175 (14)	0.0120 (9)	−0.0016 (10)	−0.0014 (10)
O1A	0.0195 (7)	0.0220 (7)	0.0242 (8)	0.0083 (5)	0.0007 (5)	−0.0015 (6)
C7A	0.0205 (9)	0.0276 (10)	0.0291 (9)	0.0101 (8)	0.0000 (7)	−0.0028 (7)
C8A	0.0302 (10)	0.0264 (10)	0.0269 (13)	0.0130 (8)	0.0012 (10)	−0.0008 (10)
Cl1A	0.0536 (7)	0.0319 (4)	0.0280 (6)	0.0127 (4)	0.0008 (4)	−0.0042 (4)
C5C	0.0183 (8)	0.0229 (7)	0.0180 (7)	0.0063 (6)	−0.0032 (6)	−0.0011 (5)
C6C	0.024 (3)	0.027 (3)	0.018 (3)	0.014 (2)	−0.001 (3)	0.004 (2)
O1C	0.031 (2)	0.0221 (16)	0.0192 (17)	0.0131 (15)	0.0025 (15)	0.0020 (13)
C7C	0.034 (2)	0.025 (2)	0.023 (2)	0.015 (2)	−0.0030 (18)	0.0020 (18)
C8C	0.030 (2)	0.022 (2)	0.020 (3)	0.0110 (17)	−0.001 (2)	−0.001 (2)
Cl1C	0.0540 (17)	0.0357 (13)	0.0318 (16)	0.0149 (11)	−0.0056 (10)	0.0022 (11)
O2B	0.0167 (4)	0.0348 (5)	0.0172 (4)	0.0065 (3)	−0.0011 (3)	0.0034 (3)
O3B	0.0211 (4)	0.0440 (5)	0.0184 (4)	0.0123 (4)	0.0074 (3)	0.0032 (4)
N1B	0.0132 (4)	0.0191 (4)	0.0164 (4)	0.0043 (3)	0.0012 (3)	0.0001 (3)
N2B	0.0164 (4)	0.0204 (5)	0.0165 (4)	0.0034 (4)	0.0008 (3)	0.0037 (4)
N3B	0.0161 (4)	0.0222 (5)	0.0178 (5)	0.0058 (4)	0.0027 (3)	0.0033 (4)
C1B	0.0139 (5)	0.0174 (5)	0.0163 (5)	0.0034 (4)	−0.0006 (4)	−0.0001 (4)
C2B	0.0135 (5)	0.0168 (5)	0.0171 (5)	0.0037 (4)	0.0007 (4)	0.0014 (4)
C3B	0.0168 (5)	0.0182 (5)	0.0180 (5)	0.0044 (4)	0.0005 (4)	0.0027 (4)
C4B	0.0194 (6)	0.0374 (7)	0.0232 (6)	0.0070 (5)	−0.0022 (5)	0.0067 (5)
C5B	0.0146 (6)	0.0227 (18)	0.0201 (10)	0.0068 (13)	0.0032 (6)	−0.0026 (16)
C6B	0.0210 (17)	0.0255 (12)	0.0212 (17)	0.0004 (11)	0.0067 (11)	0.0031 (11)
O1B	0.0187 (7)	0.0266 (8)	0.0339 (10)	0.0004 (6)	0.0045 (7)	−0.0036 (7)
C7B	0.0209 (10)	0.0336 (13)	0.0343 (11)	−0.0001 (9)	0.0051 (8)	0.0001 (9)
C8B	0.0219 (14)	0.0274 (14)	0.0350 (19)	−0.0007 (10)	0.0009 (14)	0.0049 (14)
Cl1B	0.0289 (8)	0.0350 (5)	0.0429 (2)	0.0056 (4)	−0.0032 (5)	0.0020 (3)
C5D	0.0146 (6)	0.0227 (18)	0.0201 (10)	0.0068 (13)	0.0032 (6)	−0.0026 (16)
C6D	0.019 (2)	0.035 (2)	0.018 (3)	0.0084 (17)	0.0032 (18)	0.0055 (19)
O1D	0.0180 (12)	0.0181 (12)	0.0161 (12)	−0.0006 (9)	−0.0018 (9)	0.0082 (9)
C7D	0.0276 (18)	0.0176 (17)	0.0355 (18)	−0.0008 (14)	0.0101 (14)	0.0101 (14)
C8D	0.028 (2)	0.023 (2)	0.044 (4)	−0.0038 (18)	−0.003 (3)	0.002 (3)
Cl1D	0.0289 (8)	0.0350 (5)	0.0429 (2)	0.0056 (4)	−0.0032 (5)	0.0020 (3)

### 1-[2-(2-Chloroethoxy)ethyl]-2-methyl-4-nitro-1H-imidazole Geometric parameters (Å, º)

**Table:**

O2A—N3A	1.2336 (13)	O2B—N3B	1.2337 (12)
O3A—N3A	1.2257 (13)	O3B—N3B	1.2306 (12)
N1A—C1A	1.3617 (14)	N1B—C1B	1.3607 (14)
N1A—C3A	1.3753 (14)	N1B—C3B	1.3749 (14)
N1A—C5A	1.466 (5)	N1B—C5B	1.468 (7)
N1A—C5C	1.470 (14)	N1B—C5D	1.474 (12)
N2A—C3A	1.3197 (14)	N2B—C3B	1.3188 (14)
N2A—C2A	1.3648 (14)	N2B—C2B	1.3652 (14)
N3A—C2A	1.4286 (14)	N3B—C2B	1.4257 (14)
C1A—C2A	1.3643 (16)	C1B—C2B	1.3643 (15)
C1A—H1A	0.9500	C1B—H1B	0.9500
C3A—C4A	1.4827 (15)	C3B—C4B	1.4840 (15)
C4A—H4AA	0.9800	C4B—H4BA	0.9800
C4A—H4AB	0.9800	C4B—H4BB	0.9800
C4A—H4AC	0.9800	C4B—H4BC	0.9800
C5A—C6A	1.516 (6)	C5B—C6B	1.518 (6)
C5A—H5A1	0.9900	C5B—H5B1	0.9900
C5A—H5A2	0.9900	C5B—H5B2	0.9900
C6A—O1A	1.427 (3)	C6B—O1B	1.408 (4)
C6A—H6A1	0.9900	C6B—H6B1	0.9900
C6A—H6A2	0.9900	C6B—H6B2	0.9900
O1A—C7A	1.417 (2)	O1B—C7B	1.398 (3)
C7A—C8A	1.498 (3)	C7B—C8B	1.495 (5)
C7A—H7A1	0.9900	C7B—H7B1	0.9900
C7A—H7A2	0.9900	C7B—H7B2	0.9900
C8A—Cl1A	1.797 (3)	C8B—Cl1B	1.826 (5)
C8A—H8A1	0.9900	C8B—H8B1	0.9900
C8A—H8A2	0.9900	C8B—H8B2	0.9900
C5C—C6C	1.523 (13)	C5D—C6D	1.494 (11)
C5C—H5C1	0.9900	C5D—H5D1	0.9900
C5C—H5C2	0.9900	C5D—H5D2	0.9900
C6C—O1C	1.426 (8)	C6D—O1D	1.435 (7)
C6C—H6C1	0.9900	C6D—H6D1	0.9900
C6C—H6C2	0.9900	C6D—H6D2	0.9900
O1C—C7C	1.424 (6)	O1D—C7D	1.450 (4)
C7C—C8C	1.488 (7)	C7D—C8D	1.508 (8)
C7C—H7C1	0.9900	C7D—H7D1	0.9900
C7C—H7C2	0.9900	C7D—H7D2	0.9900
C8C—Cl1C	1.772 (6)	C8D—Cl1D	1.752 (9)
C8C—H8C1	0.9900	C8D—H8D1	0.9900
C8C—H8C2	0.9900	C8D—H8D2	0.9900
			
C1A—N1A—C3A	107.79 (9)	C1B—N1B—C3B	107.89 (9)
C1A—N1A—C5A	125.0 (5)	C1B—N1B—C5B	123.2 (5)
C3A—N1A—C5A	127.2 (5)	C3B—N1B—C5B	128.6 (5)
C1A—N1A—C5C	123.1 (13)	C1B—N1B—C5D	126.2 (10)
C3A—N1A—C5C	129.0 (13)	C3B—N1B—C5D	125.7 (10)
C3A—N2A—C2A	103.88 (9)	C3B—N2B—C2B	103.77 (9)
O3A—N3A—O2A	123.38 (10)	O3B—N3B—O2B	123.41 (9)
O3A—N3A—C2A	119.06 (10)	O3B—N3B—C2B	118.99 (9)
O2A—N3A—C2A	117.55 (9)	O2B—N3B—C2B	117.59 (9)
N1A—C1A—C2A	104.08 (9)	N1B—C1B—C2B	103.94 (9)
N1A—C1A—H1A	128.0	N1B—C1B—H1B	128.0
C2A—C1A—H1A	128.0	C2B—C1B—H1B	128.0
C1A—C2A—N2A	112.85 (10)	C1B—C2B—N2B	112.97 (9)
C1A—C2A—N3A	125.68 (10)	C1B—C2B—N3B	125.27 (10)
N2A—C2A—N3A	121.47 (10)	N2B—C2B—N3B	121.75 (9)
N2A—C3A—N1A	111.39 (9)	N2B—C3B—N1B	111.43 (9)
N2A—C3A—C4A	125.06 (10)	N2B—C3B—C4B	125.39 (10)
N1A—C3A—C4A	123.55 (10)	N1B—C3B—C4B	123.18 (10)
C3A—C4A—H4AA	109.5	C3B—C4B—H4BA	109.5
C3A—C4A—H4AB	109.5	C3B—C4B—H4BB	109.5
H4AA—C4A—H4AB	109.5	H4BA—C4B—H4BB	109.5
C3A—C4A—H4AC	109.5	C3B—C4B—H4BC	109.5
H4AA—C4A—H4AC	109.5	H4BA—C4B—H4BC	109.5
H4AB—C4A—H4AC	109.5	H4BB—C4B—H4BC	109.5
N1A—C5A—C6A	110.6 (5)	N1B—C5B—C6B	113.7 (5)
N1A—C5A—H5A1	109.5	N1B—C5B—H5B1	108.8
C6A—C5A—H5A1	109.5	C6B—C5B—H5B1	108.8
N1A—C5A—H5A2	109.5	N1B—C5B—H5B2	108.8
C6A—C5A—H5A2	109.5	C6B—C5B—H5B2	108.8
H5A1—C5A—H5A2	108.1	H5B1—C5B—H5B2	107.7
O1A—C6A—C5A	112.8 (5)	O1B—C6B—C5B	113.0 (5)
O1A—C6A—H6A1	109.0	O1B—C6B—H6B1	109.0
C5A—C6A—H6A1	109.0	C5B—C6B—H6B1	109.0
O1A—C6A—H6A2	109.0	O1B—C6B—H6B2	109.0
C5A—C6A—H6A2	109.0	C5B—C6B—H6B2	109.0
H6A1—C6A—H6A2	107.8	H6B1—C6B—H6B2	107.8
C7A—O1A—C6A	113.8 (2)	C7B—O1B—C6B	113.8 (2)
O1A—C7A—C8A	108.77 (17)	O1B—C7B—C8B	109.9 (3)
O1A—C7A—H7A1	109.9	O1B—C7B—H7B1	109.7
C8A—C7A—H7A1	109.9	C8B—C7B—H7B1	109.7
O1A—C7A—H7A2	109.9	O1B—C7B—H7B2	109.7
C8A—C7A—H7A2	109.9	C8B—C7B—H7B2	109.7
H7A1—C7A—H7A2	108.3	H7B1—C7B—H7B2	108.2
C7A—C8A—Cl1A	112.35 (16)	C7B—C8B—Cl1B	113.0 (3)
C7A—C8A—H8A1	109.1	C7B—C8B—H8B1	109.0
Cl1A—C8A—H8A1	109.1	Cl1B—C8B—H8B1	109.0
C7A—C8A—H8A2	109.1	C7B—C8B—H8B2	109.0
Cl1A—C8A—H8A2	109.1	Cl1B—C8B—H8B2	109.0
H8A1—C8A—H8A2	107.9	H8B1—C8B—H8B2	107.8
N1A—C5C—C6C	113.1 (12)	N1B—C5D—C6D	107.5 (9)
N1A—C5C—H5C1	109.0	N1B—C5D—H5D1	110.2
C6C—C5C—H5C1	109.0	C6D—C5D—H5D1	110.2
N1A—C5C—H5C2	109.0	N1B—C5D—H5D2	110.2
C6C—C5C—H5C2	109.0	C6D—C5D—H5D2	110.2
H5C1—C5C—H5C2	107.8	H5D1—C5D—H5D2	108.5
O1C—C6C—C5C	107.2 (13)	O1D—C6D—C5D	108.7 (9)
O1C—C6C—H6C1	110.3	O1D—C6D—H6D1	109.9
C5C—C6C—H6C1	110.3	C5D—C6D—H6D1	109.9
O1C—C6C—H6C2	110.3	O1D—C6D—H6D2	109.9
C5C—C6C—H6C2	110.3	C5D—C6D—H6D2	109.9
H6C1—C6C—H6C2	108.5	H6D1—C6D—H6D2	108.3
C7C—O1C—C6C	113.8 (6)	C6D—O1D—C7D	113.3 (4)
O1C—C7C—C8C	108.6 (5)	O1D—C7D—C8D	106.2 (5)
O1C—C7C—H7C1	110.0	O1D—C7D—H7D1	110.5
C8C—C7C—H7C1	110.0	C8D—C7D—H7D1	110.5
O1C—C7C—H7C2	110.0	O1D—C7D—H7D2	110.5
C8C—C7C—H7C2	110.0	C8D—C7D—H7D2	110.5
H7C1—C7C—H7C2	108.3	H7D1—C7D—H7D2	108.7
C7C—C8C—Cl1C	113.6 (4)	C7D—C8D—Cl1D	108.1 (6)
C7C—C8C—H8C1	108.8	C7D—C8D—H8D1	110.1
Cl1C—C8C—H8C1	108.8	Cl1D—C8D—H8D1	110.1
C7C—C8C—H8C2	108.8	C7D—C8D—H8D2	110.1
Cl1C—C8C—H8C2	108.8	Cl1D—C8D—H8D2	110.1
H8C1—C8C—H8C2	107.7	H8D1—C8D—H8D2	108.4
			
C3A—N1A—C1A—C2A	−0.01 (12)	C3B—N1B—C1B—C2B	0.02 (12)
C5A—N1A—C1A—C2A	178.2 (3)	C5B—N1B—C1B—C2B	−174.9 (3)
C5C—N1A—C1A—C2A	176.0 (8)	C5D—N1B—C1B—C2B	175.7 (6)
N1A—C1A—C2A—N2A	0.01 (13)	N1B—C1B—C2B—N2B	−0.18 (12)
N1A—C1A—C2A—N3A	−179.85 (10)	N1B—C1B—C2B—N3B	178.29 (10)
C3A—N2A—C2A—C1A	0.00 (13)	C3B—N2B—C2B—C1B	0.27 (12)
C3A—N2A—C2A—N3A	179.87 (10)	C3B—N2B—C2B—N3B	−178.27 (10)
O3A—N3A—C2A—C1A	177.43 (11)	O3B—N3B—C2B—C1B	−173.51 (11)
O2A—N3A—C2A—C1A	−3.03 (17)	O2B—N3B—C2B—C1B	5.93 (16)
O3A—N3A—C2A—N2A	−2.42 (17)	O3B—N3B—C2B—N2B	4.84 (16)
O2A—N3A—C2A—N2A	177.12 (10)	O2B—N3B—C2B—N2B	−175.72 (10)
C2A—N2A—C3A—N1A	−0.01 (12)	C2B—N2B—C3B—N1B	−0.25 (12)
C2A—N2A—C3A—C4A	−179.66 (11)	C2B—N2B—C3B—C4B	178.83 (11)
C1A—N1A—C3A—N2A	0.02 (13)	C1B—N1B—C3B—N2B	0.15 (13)
C5A—N1A—C3A—N2A	−178.2 (3)	C5B—N1B—C3B—N2B	174.7 (3)
C5C—N1A—C3A—N2A	−175.7 (9)	C5D—N1B—C3B—N2B	−175.6 (6)
C1A—N1A—C3A—C4A	179.67 (10)	C1B—N1B—C3B—C4B	−178.95 (11)
C5A—N1A—C3A—C4A	1.5 (4)	C5B—N1B—C3B—C4B	−4.4 (4)
C5C—N1A—C3A—C4A	3.9 (9)	C5D—N1B—C3B—C4B	5.3 (6)
C1A—N1A—C5A—C6A	−76.1 (7)	C1B—N1B—C5B—C6B	71.3 (8)
C3A—N1A—C5A—C6A	101.8 (7)	C3B—N1B—C5B—C6B	−102.5 (8)
N1A—C5A—C6A—O1A	−65.6 (8)	N1B—C5B—C6B—O1B	63.8 (10)
C5A—C6A—O1A—C7A	−81.8 (5)	C5B—C6B—O1B—C7B	88.4 (5)
C6A—O1A—C7A—C8A	−165.1 (2)	C6B—O1B—C7B—C8B	166.9 (3)
O1A—C7A—C8A—Cl1A	−66.8 (2)	O1B—C7B—C8B—Cl1B	68.2 (4)
C1A—N1A—C5C—C6C	−92.7 (19)	C1B—N1B—C5D—C6D	88.2 (13)
C3A—N1A—C5C—C6C	82 (2)	C3B—N1B—C5D—C6D	−96.8 (14)
N1A—C5C—C6C—O1C	−64 (2)	N1B—C5D—C6D—O1D	67.1 (16)
C5C—C6C—O1C—C7C	−169.2 (10)	C5D—C6D—O1D—C7D	176.3 (8)
C6C—O1C—C7C—C8C	168.8 (6)	C6D—O1D—C7D—C8D	−162.6 (6)
O1C—C7C—C8C—Cl1C	68.1 (6)	O1D—C7D—C8D—Cl1D	−69.9 (7)

### 1-[2-(2-Chloroethoxy)ethyl]-2-methyl-4-nitro-1H-imidazole Hydrogen-bond geometry (Å, º)

**Table:**

*D*—H···*A*	*D*—H	H···*A*	*D*···*A*	*D*—H···*A*
C1*A*—H1*A*···N2*B*^i^	0.95	2.61	3.5144 (15)	160
C4*A*—H4*AA*···Cl1*A*	0.98	2.91	3.888 (2)	172
C4*A*—H4*AA*···O1*C*	0.98	2.39	3.024 (4)	122
C4*A*—H4*AB*···O2*B*	0.98	2.34	3.2626 (14)	157
C5*A*—H5*A*1···O2*A*^ii^	0.99	2.65	3.562 (10)	153
C5*A*—H5*A*2···O3*B*^i^	0.99	2.51	3.202 (10)	127
C7*A*—H7*A*2···Cl1*B*^iii^	0.99	2.96	3.778 (3)	141
C8*A*—H8*A*1···O1*B*^iv^	0.99	2.42	3.338 (3)	153
C8*A*—H8*A*1···Cl1*B*^iv^	0.99	2.99	3.725 (3)	132
C8*A*—H8*A*2···N2*B*^iv^	0.99	2.63	3.419 (3)	137
C5*C*—H5*C*2···O3*B*^i^	0.99	2.31	3.16 (3)	144
C1*B*—H1*B*···N2*A*	0.95	2.65	3.5495 (14)	157
C4*B*—H4*BB*···O2*A*^v^	0.98	2.45	3.2622 (15)	140
C4*B*—H4*BC*···O1*D*	0.98	2.38	3.124 (3)	133
C5*B*—H5*B*1···O3*A*	0.99	2.53	3.199 (12)	124
C5*B*—H5*B*1···O2*B*^vi^	0.99	2.60	3.439 (10)	142
C5*B*—H5*B*2···O2*B*^vii^	0.99	2.60	3.379 (10)	135
C8*B*—H8*B*2···O1*A*^viii^	0.99	2.39	3.292 (5)	151
C5*D*—H5*D*1···O3*A*	0.99	2.53	3.26 (2)	130
C5*D*—H5*D*1···O2*B*^vi^	0.99	2.52	3.345 (19)	141
C5*D*—H5*D*2···O2*B*^vii^	0.99	2.61	3.297 (17)	127
C7*D*—H7*D*2···Cl1*C*^vii^	0.99	2.81	3.742 (6)	157

Symmetry codes: (i) *x*, *y*, *z*−1; (ii) *x*−1, *y*, *z*; (iii) *x*−1, *y*, *z*−1; (iv) −*x*+1, −*y*+1, −*z*+1; (v) *x*, *y*, *z*+1; (vi) −*x*+1, −*y*, −*z*+1; (vii) *x*+1, *y*, *z*; (viii) −*x*+2, −*y*+1, −*z*+1.
